# A Comparative Analysis of Outcomes of Conventional Cold Dissection Versus Laser Tonsillectomy in Pediatric Cases in a Tertiary Care Hospital in Haryana

**DOI:** 10.1007/s12070-020-02301-1

**Published:** 2021-01-07

**Authors:** Amit Kumar, Surender Kumar, Anand Krishnan, Manish Verma, Uma Garg, Naveen Sharma

**Affiliations:** Department of ENT & HNS, BPS GMC (W), Khanpur Kalan, Sonepat, Haryana India

**Keywords:** Chronic tonsillitis, Tonsillectomy, LASER tonsillectomy, Conventional cold dissection

## Abstract

Tonsillectomy is one of the commonest ENT procedures done in paediatric population, the technique of which has evolved over years to decrease the morbidity associated with the surgery. This prospective randomized comparative study is done to evaluate the efficacy of two different techniques of this surgery, conventional cold dissection and laser tonsillectomy based on operative time, blood loss, post-operative pain and occurrence of secondary complications. The study was done in 68 patients of paediatric age group, 34 in each group underwent cold dissection and laser tonsillectomy. Operative time and bleeding were significantly low for laser group. Pain score was comparable in early post-operatives days, but was high towards the end of first week. Our study reported only one incidence of complication in the form of a secondary bleeding.

## Introduction

Waldeyer’s ring is an aggregate of lymphoid tissue located in the nasopharynx and oropharynx at the proximal parts of the aero digestive tract [1]. Palatine tonsils, which are components of Waldeyer’s ring, are collection of lymphoid tissue situated in oropharynx within tonsillar fossa. The lymphoid tissue of the tonsils are immunologically most active during the ages of 4 and 10 years [2]. For unknown reasons, their protective mechanism fails sometime and becomes seat of infection causing throat pain, fever and other complications. Such chronic infections of tonsils demands its removal [1].

Tonsillectomy is one of the most commonly performed surgical procedure in ENT [3]. This is mainly done for chronic tonsillitis and Obstructive Sleep Apnea (OSA). Standard or extra capsular tonsillectomy, which is usually done under general anesthesia, involves surgically removing the palatine tonsil along with its capsule, and then achieving hemostasis by sealing the blood vessels with the help of ligatures (ties), sutures, or heat (diathermy) [4]. The methods of tonsillectomy is usually divided into cold and hot methods. Various methods for tonsillectomy are described in the literature which includes Guillotine method, cold knife dissection, cryosurgery, monopolar and bipolar diathermy dissection, thermal welding, ultrasonic removal, radiofrequency surgery, coblation and Light Amplification by Stimulated Emission of Radiation (LASER) surgery [5–12]. All these techniques have evolved over the years aiming at decreasing the intraoperative and postoperative morbidity of the procedure and to make it safer. This entails a shorter procedure time, minimal blood loss during surgery, minimal risk of postoperative complications- mainly secondary haemorrhage, and decreased pain.

Even though we can find many literature in this topic of evaluating and comparing different methods of tonsillectomy, the superiority of one technique over the other is still in question [13]. In comparing conventional cold dissection tonsillectomy and LASER tonsillectomy, many studies have been done in respect to the above mentioned parameters. Conventional cold dissection tonsillectomy is done with cold steel instruments and haemostasis is obtained with either ligation technique or by using electro cautery. Recently LASER has been used to reduce the intraoperative blood loss during the surgery of tonsillectomy [14]. Different LASERs used in tonsillectomy are Argon plasma coagulation (APC), Neodymium; Yttrium aluminium garnet (Nd;Yag), Potassium Titanyl Phosphate (KTP) crystal and Carbon dioxide (CO2) laser. However recent developments in technology of CO2 LASER offer advantages of tissue cutting as well as tissue ablation [15].

Though LASER offers a technologically well advanced technique, many of the hospitals and surgeons still prefer the conventional cold dissection technique. There is still inadequate evidence to determine whether LASER tonsillectomy is better than other methods of tonsillectomy. Controversial opinions are there regarding which methods of tonsillectomy is superior in view of different parameters mentioned above. This emphasizes the need of further studies on this topic. So the purpose of this study is to compare conventional cold dissection tonsillectomy and LASER tonsillectomy in terms of intra operative efficiency and post-operative morbidity, which may help us to determine a better procedure for implementing in health care centres.

## Materials and Methods

This is a prospective randomized comparative study conducted in patients with chronic adenotonsillitis or OSA undergoing tonsillectomy with or without adenoidectomy in the age group 3–15 years who were selected consecutively as and when they presented during the study period in ENT department of Bhagat Phool Singh Government Medical College for Women, Khanpur Kalan, Sonipat, Haryana.

This study involved 68 patients out of which 34 patients each were randomly allotted to two groups—Group C and Group L. Group C underwent conventional dissection tonsillectomy while Group L underwent laser tonsillectomy. This division was by computerized random number table method. A complete history, ENT examination and appropriate investigations as per proforma attached were done to arrive at the correct diagnosis. A patient information sheet containing details of the study was given and an informed and written consent was obtained from the guardians of patients who enrolled for the study.

In the conventional dissection technique of tonsillectomy, a blunt dissector was used to dissect the tonsil tissue from the tonsillar bed and tonsil snare was applied at the tissue attachment in the inferior pole. Laser tonsillectomy was performed using AcuPulse Carbon Dioxide system (Lumenis, Germany) in continuous mode at 18 watts with circular dot of size 2.0 mm. The laser was used to separate the tonsil tissue from tonsillar bed. Laser precaution procedures were adhered to. Haemostasis was secured by suture ligation or electro cautery in both the techniques.

Surgical time was measured from time of putting mucosal incision to the time of securing complete haemostasis, excluding the time for adenoidectomy if done. Intraoperative blood loss was measured by weighing the swabs before and after tonsillectomy and by measuring the amount in the suction bottle. Separate suction bottle and swabs were kept for adenoidectomy. Postoperative outcomes were obtained via answers to a survey administered to the patient or caregiver. This included a combination of the Wong-Baker FACES pain scale and a set of questions in the form of a questionnaire in order to evaluate patients return to normal diet and activity and pain level. In addition, caregivers were also asked how their daily activity was affected by their child’s recovery course. The care-givers or patient filled identical copies of this survey on Post-operative days 0, 1 and 7.

Intravenous and oral Paracetamol were the standard pain control regime used in all patients given every 8 hourly during hospital stay. Patients were discharged on 1 or 2 days after the operation. Patients were reviewed in the outpatient department after one week of surgery. Patients who failed to come for follow-up were excluded.

## Results and Analysis

### Age Comparison

Table 1 shows the age comparison among the two groups. Group C had subjects with mean age of 8.29 ± 3.20 years whereas mean age in group L was 7.79 ± 3.08 years. The difference in the mean age of the two groups was not statistically significant.

**Table 1:** Age comparison

Variable	Group C	Group L
Age	8.29 ± 3.20	7.79 ± 3.08
Range (median)	4–15 (7.50)	4–15 (7.00)

Mann Whitney test, *p* value = 0.509

### Age Distribution

**Fig. 1:**
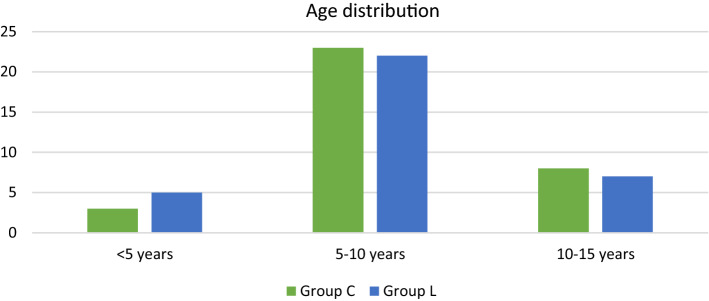
Age distribution

Table 2 shows the age distribution among the study groups. Age group of 5–10 years had maximum study participants with 67.6% and 64.7% in group C and group L respectively. Age group of 10–15 years had 23.5% and 20.6% subjects in group C and group L respectively whereas age group less than 5 years had only 8.8% and 14.7% subjects respectively in group C and group L (Fig. 1).

**Table 2:** Age distribution

Variable	Group C	Group L
< 5 years	3 (8.8%)	5 (14.7%)
5–10 years	23 (67.6%)	22 (64.7%)
10–15 years	8 (23.5%)	7 (20.6%)
Total	34	34

Chi-square test, *p* value = 0.745

### Gender Distribution

Table 3 shows the gender distribution among the two groups. In group C, males and females were 19 (55.88%) and 15 (44.12%). In group L had males and females were 18 (52.94%) and 16 (47.06%). On Chi-square test, the difference in distribution of gender in two study groups was not statistically significant (*p* value = 0.808).

**Table 3:** Gender distribution

	Group C	Group L	Total
Male	19 (55.88%)	18 (52.94%)	37 (54.41%)
Female	15 (44.12%)	16 (47.06%)	31 (45.59%)
Total	34	34	68

Chi-square test, *p* value = 0.808

### Associated Symptoms

Table 4 shows the common associated symptoms of the patients in the study population and the number and percentage of the population affected. Most common associated symptom was history of recurrent episodes of upper respiratory tract infections which was present in 54 (79.41%) patients. Other symptoms were mouth breathing in 44 (64.71%), snoring in 39 (57.35%), difficulty in breathing in 27 (39.71%) and difficulty in swallowing in 12 (17.65%) patients.

**Table 4:** Associated symptoms in study population

Associated symptoms	No. of patients affected	Percentage (%) of patients affected
Recurrent URTI	54	79.41
Difficulty in swallowing	12	17.65
Difficulty in breathing	27	39.71
Mouth breathing	44	64.71
Snoring	39	57.35

### Operating Time

Independent t test, *p* value =  < 0.001.

**Fig. 2:**
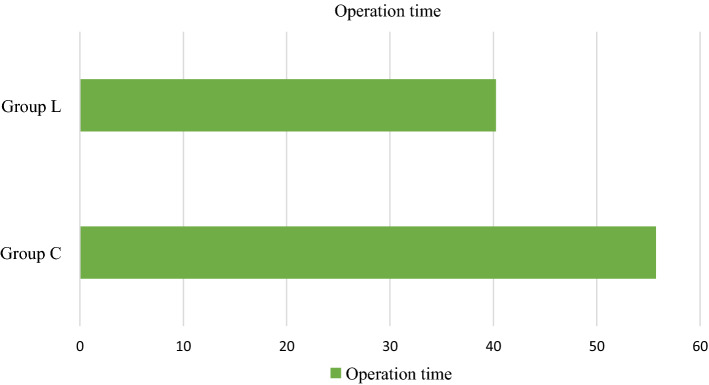
Operating time comparison between Group C & Group L

Table 5 shows the comparison of average operative time during surgery between the group C and L respectively. Operation time was higher in group C (55.74 ± 14.05 min) as compared to group L where 40.24 ± 10.79 min of operative time occurred. The median operation time was 54 min and 38 min respectively in group C and L respectively. The difference in the mean operative time was significant (*p* value =  < 0.001) (Fig. 2).

**Table 5:** Mean operation time

Variable	Group C	Group L
Mean operation time	55.74 ± 14.05	40.24 ± 10.79
Range (median)	35–98 (54.00)	24–75 (38.00)

### Intraoperative Blood Loss

**Fig. 3:**
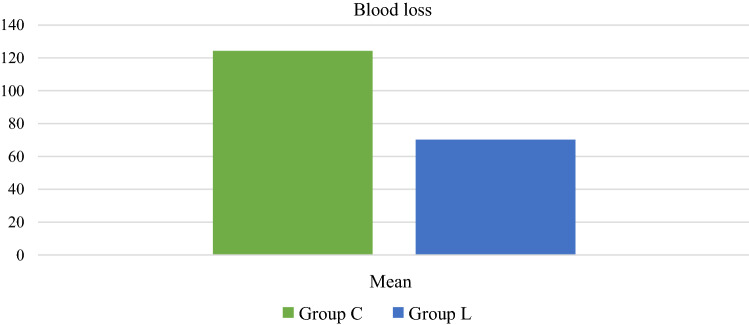
Intraoperative blood loss comparison between Group C & Group L

Table 6 shows the comparison of average blood loss during surgery between the group C and L respectively. Blood loss was higher in group C (124.15 ± 40.51 ml) as compared to group L where 70.24 ± 18.78 ml of blood loss occurred. The median blood loss was 124 ml and 68 ml respectively in group C and L respectively. The difference in the mean blood loss was significant (*p* value =  < 0.001) (Fig. 3).

**Table 6:** Blood loss comparison

Variable	Group C	Group L
Mean blood loss	124.15 ± 40.51	70.24 ± 18.78
Range (median)	57–210 (124.00)	45–130 (68.00)

Independent t test, *p* value =  < 0.001

### Post-operative Pain

**Fig. 4:**
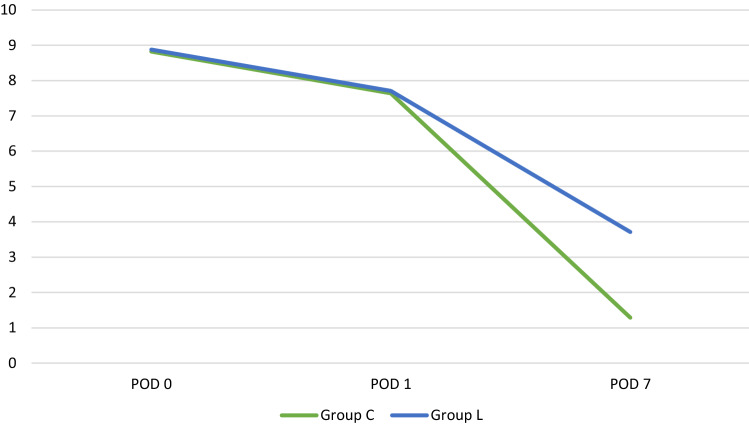
Overall pain score comparison between Group C & Group L

Table 7 shows the pain score comparison on different days after the intervention. On first post-operative day, the pain score were 9.29 ± 0.97 and 9.53 ± 0.86 in group C and group L respectively. On second post-operative day, the pain scores were 7.71 ± 1.21 and 7.71 ± 1.31 in group C and L respectively. The difference in the pain score was not significantly different between the groups. However, on seventh post-operative day, group C had significantly lower pain scores (1.29 ± 1.29) than group L (3.71 ± 1.00) (*p* value =  < 0.001) (Fig. 4).

**Table 7:** Pain score comparison between groups

Pain score	Group C	Group L	*p* value*
POD-1	8.82 ± 0.99	8.88 ± 1.01	0.808
POD-2	7.64 ± 1.25	7.71 ± 1.31	0.868
POD-7	1.29 ± 1.29	3.71 ± 1.00	< 0.001

Values in parenthesis are median. *Mann Whitney test

## Discussion

Tonsillectomy being one of the most common surgical procedures, several attempts have been done to modify the technique in order to reduce intra-operative and post-operative morbidity [4]. Several methods were tried for tonsillectomy in the past and are still evolving to achieve better results. Techniques discussed in the literature are Guillotine method, cold knife dissection, cryosurgery, monopolar and bipolar diathermy dissection, thermal welding, ultrasonic removal, radiofrequency surgery, coblation and Light Amplification by Stimulated Emission of Radiation (LASER) surgery. Many of the older techniques have been replaced by safer and newer methods. Newer techniques are concerned about decreasing the morbidity by reducing blood loss, operating time, post-operative pain, complications and improving patient comfort and oral intake following procedure. Reducing intraoperative bleeding is an important aspect especially in paediatric age group with limited blood volume and in patients with coagulopathies [16]. Reduced postoperative pain makes the patient comfortable and helps in speedy recovery. Decreased pain also helps in optimization of oral intake which in turn decreases the risk of dehydration, infection and thus postoperative complications such as delayed haemorrhage [[1].

Our study is a prospective comparative study to assess the outcomes of conventional cold dissection method and laser tonsillectomy. Conventional cold dissection is still one of the most commonly used technique for tonsillectomy worldwide with satisfactory results. Lasers are relatively new to the armamentarium of tonsil surgeons compared to cold dissection. Even though it is a newer technique, it has gained a place in the field recently due to sophistication in the machinery.

We conducted this study in 68 patients in age group 3–15 years who were scheduled to undergo elective tonsillectomy. The majority of the population who undergo tonsillectomy belong to paediatric age [17]. Blood loss of any amount is significant in children because of low blood volume [16]. Compared to adults pain management in paediatric age group is more important because it affects oral intake and overall recovery. Our study involved patients in age group of 3–15 with mean age of the patients in Group C was 8.29 ± 3.20 years and 7.79 ± 3.08 years in Group L. Statistically there was no significant difference between the mean age of two groups and hence both the groups were comparable in terms of age also. In our study, there were 19 (55.88%) males and 15 (44.12%) females in Group C and 18 (52.94%) males and 16 (47.06%) females in Group L. Thus the gender distribution was statistically comparable in both the groups, even though none of the study mention gender as a confronting factor.

Understanding the common symptomatology of chronic tonsillitis is significant for clinical diagnosis and management. The basic symptomatology varies between chronic tonsillitis and chronic adenotonsillitis. Recurrent infections of the upper respiratory tract was the most common symptom reported in 54 patients (79.41%). Symptoms related to adenoid hypertrophy like mouth breathing and snoring were present in 44 (64.71%) and 39 (57.35%) patients each. Other throat related symptoms like difficulty in swallowing and breathing were also reported.

In the present study, the operative time was recorded from time of putting the mucosal incision till the achievement of complete haemostasis. The average time required in Group C population who underwent conventional cold dissection tonsillectomy was 55.74 ± 14.05 min ranging from a minimum of 35 min to a maximum of 98 min. In Group L the average operating time was 40.24 ± 10.79 min with a range of 14–75 min. This shows a considerably low operating time for laser group which is statistically significant (*p* < 0.05). During conventional cold dissection tonsillectomy the dissection is carried out with blunt instrument which leads to more damage to the tissue and the blood vessels within the tonsillar bed leading to active bleeding in the fossa which requires more time to achieve haemostasis. On the other hand, decreased operative time in Group L can be attributed to the property of laser to dissect tissue and coagulate small blood vessels at the same time which in turn reduced the time required for haemostasis [1]. Surgical procedure associated with better haemostasis invariably leads to reduction in operative time. This particularly applies to tonsillectomy that is performed in relatively narrow constraints with limited surgical exposure and limited space for instrumentation.

In the study by Ishlah Wan et al. done in 60 patients, similar results were obtained with the mean operating time 25.23 min in laser group and 31.90 min in dissection group [1]. They proposed that the simultaneous cutting and coagulation achieved by the laser helped them to achieve a near bloodless field for surgeon to operate on smoothly and thus reducing total operating time. In the study it was concluded that the reduced operative time was significant as numbers of operations performed can be increased, hence avoiding unnecessary cancellation of surgeries in a fixed theatre session. Bergler et al. observed that the mean duration of procedure reduced by 50% with laser technique of tonsillectomy [18].

There were some studies with results contradicting that of our study. Strunk C L observed that the laser tonsillectomy is more time consuming contradicting to our results [19]. According to them the increased total operating time was a result of increased setup time and laser malfunctions. These were significant problems in early nineties when this study was conducted but not in the current scenario. And in our study the set up time was not counted in operating time.

In our study, the intraoperative blood loss was calculated, the mean blood loss was 124.15 ± 40.51 ml in Group C ranging from 57 to 210 ml. the mean blood loss in Group L was 70.24 ± 18.78 ml ranging from 45 to 130 ml. During statistical analysis, the difference in intraoperative blood loss between the two procedures was found to be significant (*p* < 0.05). Along with the sharp tissue dissection, the vessels are also precisely coagulated in laser assisted tonsillectomies. This provides a bloodless tonsillar fossa during the entire procedure. But in conventional cold dissection tonsillectomy the trauma caused to soft tissue is more due to stretching of tissue caused by blunt dissection. This leads to irregularly cut bleeding edges of the tissue along with torn vessel walls leading to a bleeding fossa after the removal of tonsil tissue. It requires a cumbersome haemostatic procedure to achieve a completely blood less tonsillar fossa.

The lesser blood loss in Group L makes it a preferred method especially in children where the blood volume is low and even the small amount of blood loss is significant. Moreover tissue loss and soft tissue trauma are expected to be less whenever blood loss is less. Thus the blood loss is considered an indirect indicator of precision in dissection also.

Most of the previous studies in this field also found results similar to our study. In a study done in 201 patients, Bergler et al. observed that the average blood loss in laser group was 13.9 ml compared to 131.9 ml in cold dissection group. They inferred that the blood loss was decreased by 90% with laser procedure compared to conventional cold dissection procedures [18]. In a study conducted by Linder et al. exclusively in paediatric population as in our study, the results were also similar. The mean age of the study population was 5 years. This study suggested that laser achieves better haemostasis which is very helpful especially in paediatric population [20].

Ishlah Wan et al. observed that the evidence of endothelial damage and thrombosis of the capillaries and small diameter vessel in the immediate vicinity of laser wound which might have caused better haemostasis in laser procedure [1].

Auf et al. stated that laser technique had only a marginal advantage over conventional method in case of intra operative blood loss, but a high incidence of secondary haemorrhage was associated with laser technique [21]. The increased secondary haemorrhage was attributed to increased necrotic tissue due to excessive thermal injury caused by laser dissection directed towards tonsillar bed. Our study did not report any such incidence of reactionary or secondary bleeding with laser procedure owing to precise coagulation of the bleeding vessels and judicious use of laser during dissection.

In the present study the post-operative pain is measured using Wong-Baker FACES pain scale as it gives more sensitive and precise measurements than the other descriptive scales. Postoperatively analgesia was provided with oral/intravenous Paracetamol according to weight (10–15 mg/Kg). Various analgesics for postoperative pain relief was used in different studies. Linder et al. used Paracetamol while Ishlah Wan et al. used tramadol and Bergler et al. used a combination of Paracetamol and diclofenac [1,[ 18,[ 20] like morphine and other opioids. The reason for choosing Paracetamol as the drug for providing postoperative supplementation of analgesia was that its elimination half-life (± ½) was less than 12 h. Therefore it was less likely to affect the subsequent value of the pain score which was thought to be appropriate with the time intervals chosen for postoperative pain assessment.

In our study, pain score was recorded on POD 0, 1 and 7. On POD 0, pain score average was 8.82 ± 0.99 in Group C and 8.88 ± 1.01 in Group L with *p* value 0.808. On POD 1, pain score average was 7.64 ± 1.25 in Group C and 7.71 ± 1.31 in Group L with *p* value 0.868. There was no statistically significant difference between both groups in the evaluation of pain in the initial days. But significant difference was found in pain score average of POD 7 between the two groups, which were 1.29 ± 1.29 in Group C and 3.71 ± 1.00 in Group L with *p* value < 0.05.

The aim of reducing the postoperative pain is not only the patient comfort, but also improving the oral intake to reduce risk of dehydration, infection and secondary haemorrhage [1]. In the initial days (POD 0 and 1), the causes of pain in conventional cold dissection is due to increased manipulation and stretching of the tissue in tonsillar fossa, which is not present in laser dissection [22]. Although the pain due to thermal injury is more in laser group, the temporary desensitisation effect of the laser on nerve endings causes decreased sensation of this pain in initial days. This results in a comparatively similar pain scores in both the groups in the early post-operatives days.

Towards the end of the first post-operative week the pain scores decrease continuously in conventional cold dissection group while this is hampered in laser group by several reasons creating a significant difference between the two. Auf et al. stated that the laser has an immediate desensitising effect on the cut ends of the nerve endings initially which is lost in following days causing increased pain sensation. They also observed that the thermal injury caused by the laser takes more time to heal and forms a thick slough layer in the tonsillar bed causing delayed contraction and pain [21]. Chances of formation of secondary infections under these thick slough are also to be considered.

Oas et al. observed that the wound crater created by laser shows three distinctive layers of tissue. Firstly a layer of carbonisation containing amorphous burned materials, second layer of necrotic cells and third layer of oedematous surround tissue. The amount of scattered laser determines the amount of thermal injury in surrounding tissue which in turn determines the thickness of the necrotic layer. According to him the laser causes not only the coagulation of blood vessels but the sealing of lymphatics and nerve endings which results in initial low pain sensation. Subsequently in the second week, the thick necrotic layer act as a substrate for development of infections causing delayed healing and increase in pain [22]. Saito et al. also noticed a comparatively low pain in immediate post-operative days in laser group which gradually increased to overtake conventional cold dissection group towards post-operative days 5–8 [23]. Martin et al. also arrived at similar results of increasing pain towards the post-operative day 5 or 6 owing to development of thick slough after laser assisted tonsillectomy [24].

But there are studies showing overall decreased pain scores for laser tonsillectomy group irrespective of time. Gofman et al. found that a decreased pain helps to initiate oral intake early in laser tonsillectomy group [25]. Study conducted by Martinez et al. also found laser procedure as less painful compared to conventional tonsillectomy [24]. Ishlah et al. found comparable pain scores in laser and conventional cold dissection groups [1].

Linder et al. did a study similar to our study. The study population was of paediatric age group and the pain management was done using Paracetamol itself. They found a comparatively decreased pain scores in laser tonsillectomy than expected after conventional cold dissection [20].

No other post-operative complication occurred in our study except for secondary bleeding in one patient in Group C. Haemorrhages associated with tonsillectomy are generally classified in to three types. Primary haemorrhage is intraoperative, reactionary haemorrhage is bleeding post-operatively within 24 h and secondary haemorrhage is beyond 24 h [26]. Reactionary haemorrhages are mostly due to slippage of ligatures, failure to ligate all vessels, retention of clots leading to improper contraction of fossa, increase in blood pressure following recovery from anaesthesia especially in hypotensive anaesthesia and bleeding from injured muscles. Secondary haemorrhages are mostly due to setting up of infection in the post-operative tonsillar fossa [22].

According to severity of the bleeding, the management of post-operative bleeding changes. The patient with secondary haemorrhage in our study was readmitted in ward, investigated with blood counts and coagulation profile, started on intravenous antibiotics and was considered for re-examination under general anaesthesia. Retained thick slough was removed and bleeding points were cauterised. Post-operative period was uneventful and was discharged on oral antibiotics.

As already described, the thick slough formed by the necrotic tissue left behind in the tonsillar fossa following laser application will act both as a substrate and a cover for infection to arise. These small infections coalesce to develop a proper tonsillar fossa infection and may initiate secondary bleeding. Judicious use of antibiotics in the post-operative period is suggested many surgeons anticipating this complication.

## Conclusion

Compared to the conventional cold dissection tonsillectomy, laser assisted tonsillectomy has shown to have significantly shortened the operative time and reduces blood loss. Pain scores were comparable with cold dissection in initial post-operative days. But, towards the end of first post-operative week there was a statistically significant high pain score for laser group. We could not observe any high incidence of complications in both groups, except for a secondary haemorrhage in a single patient in conventional cold dissection group. All these results helped us to conclude that the precise dissection and coagulating effect of laser makes it a less time consuming procedure with minimal blood loss. But the low pain scores in conventional cold dissection helps in better recovery of patients with early initiation of oral intake. With the advent of newer methods like laser, electro cauterisation and usage of appropriate antibiotics, the occurrence of complications after tonsillectomy has reduced to minimal level.
